# Development of Glioblastoma Multiforme in Patients With Human Papillomavirus‐Positive Oropharyngeal Squamous Cell Carcinoma: A Case Series

**DOI:** 10.1002/cnr2.70521

**Published:** 2026-03-20

**Authors:** Jessica T. Lovett, Michael Wotman, Ryan Denu, Aishwarya Mandava, Hardik Shah, Robert Sebra, William Westra, Marshall Posner

**Affiliations:** ^1^ Department of Internal Medicine NYU Grossman School of Medicine New York New York USA; ^2^ Division of Cancer Medicine The University of Texas MD Anderson Cancer Center Houston Texas USA; ^3^ Department of Genetics and Genomic Sciences Icahn School of Medicine at Mount Sinai New York New York USA; ^4^ Department of Anatomic Pathology Moffitt Cancer Center Tampa Florida USA; ^5^ Tampa General Hospital Cancer Institute/Cancer Center of South Florida Tampa Florida USA

## Abstract

**Background:**

Human papillomavirus‐positive oropharyngeal squamous cell carcinoma (HPV+ OPC) patients typically exhibit reduced rates of second primary malignancies (SPMs) compared to HPV‐negative head and neck cancer patients. While HPV+ OPC patients may be predisposed to SPMs in HPV‐associated anatomical sites, the metachronous presentation of an HPV+ OPC and subsequent glioblastoma multiforme (GBM) has not been previously documented.

**Cases:**

We present two cases of HPV+ OPC patients who developed GBMs within a short period after definitive therapy.

**Methods:**

Comprehensive whole exome sequencing (WES) was performed on paired GBM, oropharyngeal, and matched normal tissues from two HPV+ OPC patients who developed GBMs within a short period after definitive therapy, with the goal of identifying shared somatic and germline variants. Bioinformatic analyses included variant calling, annotation, and pathway enrichment.

**Results:**

WES revealed a shared *CEP104* missense mutation among both oropharyngeal tumors as well as a potential *KIR2DL4* germline variant in the second patient, suggesting a possible role for disrupted NK cell immunity in driving these cancers.

**Conclusion:**

Although these cases were likely random events, they illustrate the diagnostic and therapeutic challenges of GBM following HPV+ OPC and underscore the importance of personalized genomic assessment in HPV+ OPC survivors, who may develop non‐head and neck SPMs unrelated to field cancerization.

## 1 Introduction

Human papillomavirus‐positive oropharyngeal squamous cell carcinoma (HPV+ OPC) is biologically distinct from traditional, environmentally related head and neck squamous cell carcinomas (ERHNC), differing in its pathogenesis, affected demographic, and response to treatment [1, 2, 3, 4, 5]. In contrast to patients with ERHNC, patients with HPV+ OPC are less likely to have a history of significant alcohol and tobacco consumption, resulting in lower rates of field cancerization [6]. They also exhibit decreased rates of synchronous and metachronous second primary malignancies (SPMs) in anatomical locations not traditionally associated with HPV infection [1, 2, 3, 4, 7, 8, 9]—particularly in the lung, esophagus, and other head and neck subsites [10, 11]—and are associated with significantly improved long‐term survival [12, 13, 14, 15].

Given the continued rise in HPV+ OPC and its significantly improved survival compared with ERHNC, there is an emerging need to understand long‐term sequela, particularly as it relates to the development of SPMs beyond the head and neck. Population‐based studies demonstrate that HPV+ OPC survivors exhibit fewer SPMs overall yet may remain predisposed to subsequent malignancies at HPV‐associated anatomical sites such as the cervix, vulva, and anus. Despite this, the metachronous development of glioblastoma multiforme (GBM) following HPV+ OPC has not been previously documented. Only one prior report described synchronous presentation of both malignancies [16]. Given the rarity of this occurrence and the growing population of HPV+ OPC survivors, exploring possible etiologic mechanisms is clinically relevant.

In this case series, we describe two patients who developed GBM within a short period after definitive therapy for HPV+ OPC. Given the rarity of metachronous GBM following HPV+ OPC, Whole Exome Sequencing (WES) was performed on GBM, oropharyngeal, and normal tissues from both patients to explore potential shared somatic or germline alterations that might suggest a common pathogenic mechanism.

The following is a retrospective HIPAA‐compliant case series of two HPV+ OPC patients who developed GBMs within a short period after definitive therapy and were treated at the Icahn School of Medicine at Mount Sinai. All treatment was completed by 2022.

### 1.1 Case 1

#### 1.1.1 Presentation

A 63‐year‐old male with a history of basal cell carcinoma presented with a painless, right‐sided neck mass. He was otherwise asymptomatic. He occasionally consumed alcohol but never used tobacco. He had no significant family history of cancer.

#### 1.1.2 Diagnostic Workup

CT neck revealed a 3.1 cm mass within the right jugulodigastric region and enlargement of the right base of tongue (BOT). Fine needle aspiration (FNA) of the neck mass revealed poorly differentiated carcinoma. Initial examination was notable for a mobile neck mass and well‐circumscribed area of prominence with papillations at the right BOT. He underwent another FNA, which showed p16+/HPV33+ squamous cell carcinoma (SCC). Staging PET/CT confirmed locally‐regionally advanced disease without distant metastases.

#### 1.1.3 Therapeutic Intervention

The patient enrolled in a clinical trial of surgery and de‐escalated adjuvant therapy with dose‐reduced radiation. He underwent transoral robotic surgery (TORS) and bilateral selective neck dissections. Pathology revealed a 4.2 cm BOT SCC with extensive perineural invasion and metastasis to one lymph node with extranodal extension. Final staging was pT3 pN2a cM0 (IVA) based on the American Joint Committee on Cancer 7th edition, which was in use at the time of initial staging and treatment. This was followed by chemoradiation with 56 Gy and concurrent cisplatin. A 3‐month re‐staging PET/CT showed no evidence of residual disease.

#### 1.1.4 Follow‐Up and Outcomes

The patient remained disease‐free for 2.5 years, at which point he reported new word‐finding difficulties and memory problems. He underwent an MRI brain which revealed a 4.4 cm left frontotemporal mass causing edema and midline shift. Pathology from a left temporal craniotomy and tumor resection revealed glioblastoma, WHO grade IV, IDH‐wild type, MGMT‐methylated. The patient received adjuvant partial‐brain radiotherapy (PBRT) and temozolomide (TMZ) and was enrolled in a clinical trial of tumor treatment fields and a personalized mutation‐derived tumor vaccine. Nineteen months after resection, MRI brain revealed increased enhancement in the left temporal lobe and thalamus; biopsy confirmed recurrent/residual GBM. TMZ was re‐challenged, but the recurrent tumor was found to be MGMT‐unmethylated. Therapy was switched to bevacizumab and nivolumab, with continuation of the investigational vaccine. His disease progressed, and he died 34 months after initial GBM diagnosis.

### 1.2 Case 2

#### 1.2.1 Presentation

A 57‐year‐old female presented with a right neck mass and throat discomfort. She was a former smoker with a 7.5 pack‐year history and rarely consumed alcohol. Aside from colon adenocarcinoma in the patient's father, there was no other significant family history of cancer.

#### 1.2.2 Diagnostic Workup

CT neck revealed right greater than left enlargement of the BOT and enlarged right‐sided jugulodigastric lymph nodes. FNA was inconclusive. Initial examination was notable for a right BOT mass and palpable right‐sided level II/III lymph nodes. Due to the known limitations of p16 immunostaining on FNA specimens [17] and the well‐described discordance between p16 and HPV DNA or RNA status in patients with oropharyngeal cancer [18], PCR‐based HPV genotyping was performed per institutional protocol, which identified HPV16 positivity. Repeat FNA revealed p16‐/HPV16+ SCC. Staging PET/CT confirmed local‐regionally advanced disease without distant metastases.

#### 1.2.3 Therapeutic Intervention

The patient enrolled in a pre‐operative vaccine trial. She then underwent TORS and bilateral selective neck dissections. Pathology revealed a 3 cm BOT SCC with metastasis to one 4 cm lymph node. Final staging was pT2 pN2a cM0 (IVA), per AJCC 7th edition, which was in use at the time of initial staging and treatment. This was followed by adjuvant radiation therapy with 60 Gy in 30 fractions. Her course was complicated by poor oral intake and significant weight loss requiring percutaneous endoscopic gastrostomy (PEG) tube placement. Re‐staging imaging showed no evidence of residual disease.

#### 1.2.4 Follow‐Up and Outcomes

Two years after completion of therapy, she remained disease‐free but developed new left‐sided arm and facial weakness. MRI brain revealed a 3 cm right frontal lobe mass with edema and midline shift. Pathology from her subsequent craniotomy and tumor resection revealed glioblastoma, WHO grade IV, IDH‐wild type, MGMT‐unmethylated. A follow‐up MRI brain for radiation planning was concerning for disease progression. She was started on TMZ, followed by PBRT. Her course was complicated by dysphagia requiring a second PEG tube, a lower extremity DVT requiring IVC filter placement, and seizures. After completion of adjuvant therapy, the patient's functional status rapidly declined, and she became completely dependent on others for her activities of daily living. Subsequent MRI brain revealed further disease progression with midline shift, uncal herniation, and hydrocephalus. The patient was enrolled in hospice and expired 3 months after initial GBM diagnosis.

## 2 Methods

### 2.1 Whole Exome Sequencing

Whole Exome Sequencing (WES) was performed on GBM, oropharyngeal, and normal tissues from both patients to explore potential shared somatic or germline alterations that might suggest a common pathogenic mechanism. DNA was extracted from six FFPE‐derived tissue samples. Library preparations were performed using Agilent SureSelect XT LI/HS v7 whole exome capture panel. Libraries were sequenced on an S1300 flowcell on the NovaSeq 6000 platform using a 150 nt paired‐end read configuration.

### 2.2 Data Pre‐Processing

Raw basecalls were demultiplexed using *bcl2fastq* (v2.20.0) to generate FASTQ files using default parameters. The Illumina *DRAGEN* (v3.10.4) whole exome sequencing pipeline was used for quality control and somatic variant calling. Paired‐end sequencing reads were mapped to the human reference genome. Using the *DRAGEN* somatic mode, the tumor and normal samples were mapped and aligned separately, generating their respective BAM files. The mapping rate was > 99% across the samples, with an average target coverage > 550X, and an estimated usable coverage > 290×. More than 98% of sites in the target region had ≥ 10× coverage, > 96% of sites had ≥ 50× coverage, and > 94% of sites had ≥ 100× coverage.

### 2.3 Somatic Variant Calling and Downstream Analysis

The tumor‐normal pipeline was used in *DRAGEN* for somatic variant calling to jointly analyze the tumor/normal samples and exclude germline variants generating VCF and hard‐filtered VCF files specific to tumor mutations. Unique and intersecting variants were fetched from the hard‐filtered VCF files using *bcftools isec* [19] (v1.9). VCF files were annotated using AnnoVar [20] (2019 Oct24 release). Downstream analysis was performed with R/Bioconductor *maftools* package [21].

### 2.4 Ethical Approval and Data Handling

The study was performed following institutional review board approval from the Icahn School of Medicine at Mount Sinai. Informed consent for publication of all case details and accompanying images was obtained from the next of kin of both patients, who are deceased, in accordance with institutional and journal ethical standards. Genomic data were processed and de‐identified prior to analysis to ensure patient confidentiality.

## 3 Results

Whole exome sequencing was performed on GBM, BOT, and normal tissues from patient 1 (GBM1, BOT1, NL1) and patient 2 (GBM2, BOT2, and NL2). Table 1 shows the distribution of somatic variants across the brain and BOT samples. Aside from a shared Q43R missense mutation in the KIR2DL4 gene (rs867586202) between BOT2 and NL2, there were no other common variants between samples from the same patient.

**TABLE 1:** Distribution of somatic variants across patient samples.

Sample	Patient 1 GBM	Patient 1 BOT	Patient 2 GBM	Patient 2 BOT
Unique variants	125	748	121	411
Synonymous SNV	29	231	42	125
Non‐synonymous SNV	77	448	67	256
Transitions	91	715	85	359
Transversions	29	31	29	47
Missense mutation	77	448	67	256
Nonsense mutation	7	49	2	14
Silent	29	231	42	125

*Note:* Variant counts and classifications were generated using the Illumina DRAGEN whole‐exome somatic variant calling pipeline (v3.10.4) [22].

Abbreviations: BOT, base of tongue; GBM, glioblastoma multiforme; SNV, single nucleotide variant.

While investigating the TCGA cohort [23] oncogenic signaling pathways enriched in the gene lists generated by functional annotation of somatic variants in samples from patient 1, the PTEN gene from GBM1 and AKT3 and MLST8 genes from BOT1 overlapped with the PI3K pathway. For patient 2, the PTEN, NPRL3, and PIK3R1 genes in GBM2 and MAPKAP1, MTOR, and PIK3CA genes in BOT2 overlapped with the PI3K pathway. The KSR1 and PIN1 genes in GBM2 and PDGFRB gene in BOT2 overlapped with the RTK‐RAS pathway.

At the gene‐level, three genes overlapped with somatic missense mutations at different loci between GBM1 and BOT1, including ABCB11, ATAD2B, and MYH15 genes. In ABCB11, the amino acid changes were H608R and R1268W in GBM1 and BOT1, respectively. Four genes overlapped at the gene‐level with somatic missense mutations at different loci between GBM2 and BOT2, including FAS, FLG, KCP, and LUC7L. In FAS, the amino acid changes were R71K and N234S in GBM2 and BOT2, respectively.

Samples of the same tissues were also compared. No common variants were identified between the two GBMs. One missense mutation, rs113174854, was identified between the two BOT samples with R280H in centrosomal protein 104 (CEP104 gene).

## 4 Discussion

HPV+ OPC is characterized by its distinct biologic profile, high radiosensitivity, and favorable prognosis when compared with HPV‐negative HNSCC. The occurrence of GBM as a metachronous malignancy in this population is exceedingly rare, and no mechanistic or epidemiologic association between HPV infection and GBM has been established.

**FIGURE 1:**
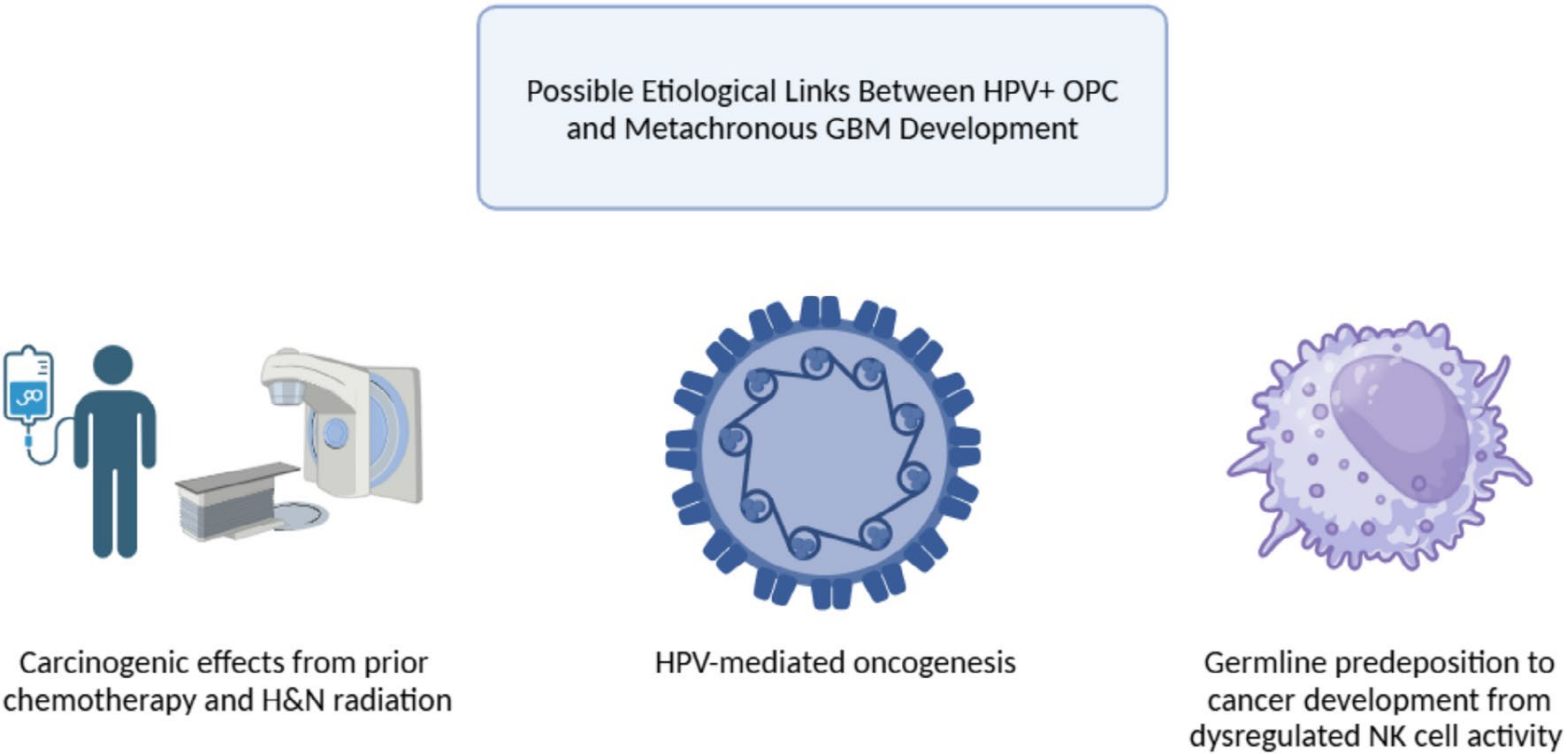
Possible etiological links between HPV+ OPC and metachronous GBM development. Abbreviations: GBM, glioblastoma multiforme; HPV+ OPC, Human papillomavirus‐positive oropharyngeal squamous cell carcinoma; H&N, head and neck; NK, natural killer. Created in https://BioRender.com.

Though several studies have proposed that HPV DNA or proteins may be detectable in gliomas, such findings are inconsistent and limited by methodological variability. Specifically, studies from Pakistan [24], Tunisia [25], the United States [26], Italy [27], and Japan [28] have documented viral nucleic acid and/or protein in glial tissue. In one study, tissue samples from patients with GBM primaries and without a history of head and neck or other HPV‐associated neoplasms were molecularly tested for HPV positivity; 28% of GBM samples were HPV+, with a signal toward longer survival [24]. Other larger studies demonstrated a more tenuous relationship; for example, HPV DNA was detected in 4 out of 514 low grade primary glioma samples in a study utilizing next‐generation sequencing to assess tumor tissue viromes [26]. While these isolated reports have identified HPV DNA or protein in GBM tissue, their biological significance remains uncertain and additional studies are warranted to further explore and validate this relationship (Figure 1). Given that HPV does not disseminate hematogenously, we did not perform additional HPV assays, as the index oropharyngeal infections were not expected to be mechanistically related.

Before investigating whether shared germline susceptibility might underlie the co‐occurrence of these malignancies, we first considered other potential causative or associative factors that could be driving this relationship. In the cases present, a hereditary cancer syndrome was not considered to be a likely causative factor as neither patient had a significant family history of cancer nor was diagnosed at a young age. We also considered the possibility that the carcinogenic effects of therapy received for the index oropharynx cancer predisposed to subsequent GBM development. Ju et al. reported the case of a 50‐year‐old male with extensive tobacco and alcohol use who developed a spinal cord GBM 7 years after completion of radiation therapy for OPC (HPV unknown) [29]. However, unlike this case, the GBMs in our study did not meet Cahan and Liwnicz's criteria for radiation‐induced CNS malignancy as they were intracranial and not located in the previously irradiated fields. Moreover, platinum agents are not associated with the development of brain neoplasms.

Given the rarity of metachronous GBM following HPV+ OPC and the absence of other plausible linking etiologies, WES was performed to identify potential shared somatic or germline alterations that might indicate a common pathogenic mechanism. To date, genome‐wide association studies (GWAS) have not identified overlapping germline risk alleles between GBM and HPV‐associated HNSCC. None of the variants detected in our patients have been previously linked to either cancer type.

As highlighted, WES of tumor and normal tissues identified two notable genetic alterations. A possible germline missense variant in the *KIR2DL4* gene (Q43R) was observed in patient two's normal and oropharyngeal tumor tissues. Second, the oropharyngeal tumors in both patients harbored a somatic *CEP104* R280H mutation, a centrosomal gene implicated in microtubule dynamics and genomic stability. Though the lack of shared somatic mutations in the GBM specimens makes it unlikely that these alterations represent a new epidemiological finding, these cases highlight the diagnostic and therapeutic challenges associated with GBM after index HNC as well as the importance of performing genetic testing in this patient population.

A shared Q43R missense mutation in the *KIR2DL4* gene (rs867586202) between the OPC and normal tissues of the second patient suggests a potential germline variant, with possible impact on the role of impaired natural killer (NK) cell function in tumorigenesis. Killer cell immunoglobulin‐like receptors (KIRs) are widely expressed on the surface of natural killer (NK) cells and, to a lesser extent, on cytotoxic T lymphocytes [30, 31]. The KIR‐L series, including KIR2DL1, KIR2DL3, and KIR2DL4, function primarily as inhibitory receptors that dampen NK cell activity. Though the specific Q43R mutation present in our patient has not been previously reported [32], dysregulation in the NK cell cytotoxicity pathway has been implicated in the development of multiple cancers [33, 34]. Although this finding is hypothesis generating and suggests a possible pathogenic role of disrupted NK cell immunity in the second case, it is unclear why the variant was not identified in the corresponding GBM specimen (Figure 1).

WES also identified a common missense mutation, *CEP104* R280H (rs113174854), in both oropharyngeal tumors. CEP104 encodes a component of the microtubule organizing center or centrosome, and germline alterations are associated with Joubert syndrome [35], though this particular alteration has not been explicitly associated with this neurodevelopmental disorder. Neither patient was expected to have this rare syndrome, of which cancer development is not a characteristic feature. Nevertheless, centrosome alterations have been extensively studied in many cancer types and are associated with genomic instability [36]. Specifically, studies have demonstrated that centrosomes of breast adenocarcinoma cells generally display an abnormal structure, aberrant protein phosphorylation, and increased microtubule nucleating capacity in comparison to centrosomes of normal epithelial and stromal tissues [37]. Therefore, somatic *CEP104* mutations may have played a role in the pathogenesis of both oropharyngeal neoplasms, which requires further investigation. However, they do not provide a mechanistic explanation for metachronous GBM development. Of note, no common somatic variants were identified between the two GBM specimens.

These cases illustrate the rare co‐occurrence of HPV+ OPC and GBM. Though patients with HPV+ OPC may have a higher risk of developing HPV‐related SPMs in anatomical locations associated with HPV infection, such as the cervix, vulva, and anus [5, 11, 38], as well as second oropharyngeal cancers associated with multifocal infection of Waldeyer's ring [39, 40, 41, 42], discussion of metachronous SPM development beyond sites associated with HPV and the upper aerodigestive tract has been more limited and has focused primarily on colon adenocarcinoma and melanoma [42, 43, 44, 45, 46]. In fact, there is only one published report of a patient with both HPV+ OPC and GBM, though the presentation was synchronous rather than metachronous [16]. This study was thus the first to perform genetic testing via WES of OPC, GBM, and normal tissues from the same patient to identify common somatic variants and potential germline predispositions.

Given the rising incidence and improved survival outcomes of HPV+ OPC, oncologists will face an increasing population of survivors. GBMs and other aggressive SPMs may become more common than previously recognized and can have profound implications. While these cases likely represent random events rather than a new epidemiological finding, they illustrate the diagnostic and therapeutic challenges associated with GBM after index HNC and the importance of performing genetic testing to better understand the association, if present, between primary and second primary neoplasms. Our study aims to better define the pattern of development of SPMs in HPV+ OPC and highlights the need for additional larger studies to further investigate the etiological link between these two neoplasms.

## Author Contributions

All authors have made substantial contributions to the conception and design of the study, as well as the analysis of data, drafting and reviewing the work for content, participating in the final approval of the version to be published, and agreeing to be accountable for all aspects of the work in ensuring that the integrity of the work is appropriately investigated and resolved.

## Funding

The study was supported by internal philanthropic funds from the Icahn School of Medicine at Mount Sinai.

## Conflicts of Interest

The authors declare no conflicts of interest.

## Data Availability

The authors declare that they had full access to all of the data in this study and the authors take complete responsibility for the integrity of the data and accuracy of the data analysis. The data supporting the findings of this study are available from the corresponding author upon reasonable request, with appropriate institutional approvals.
